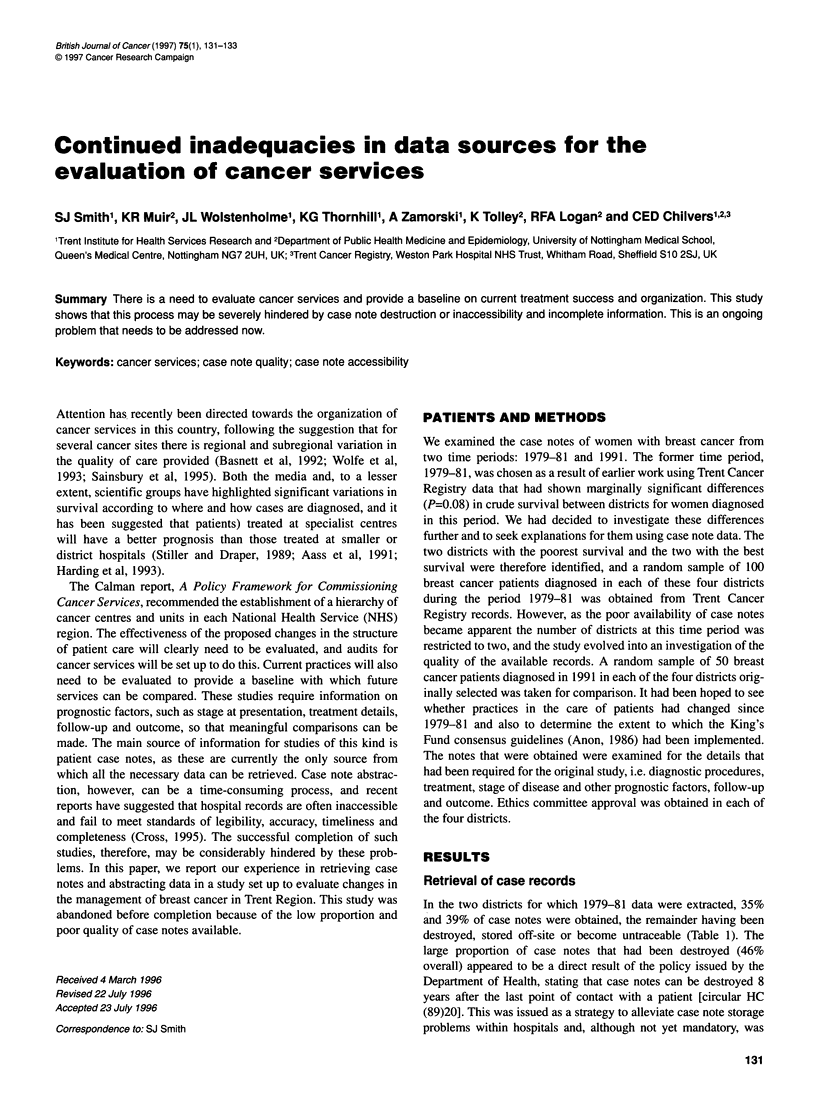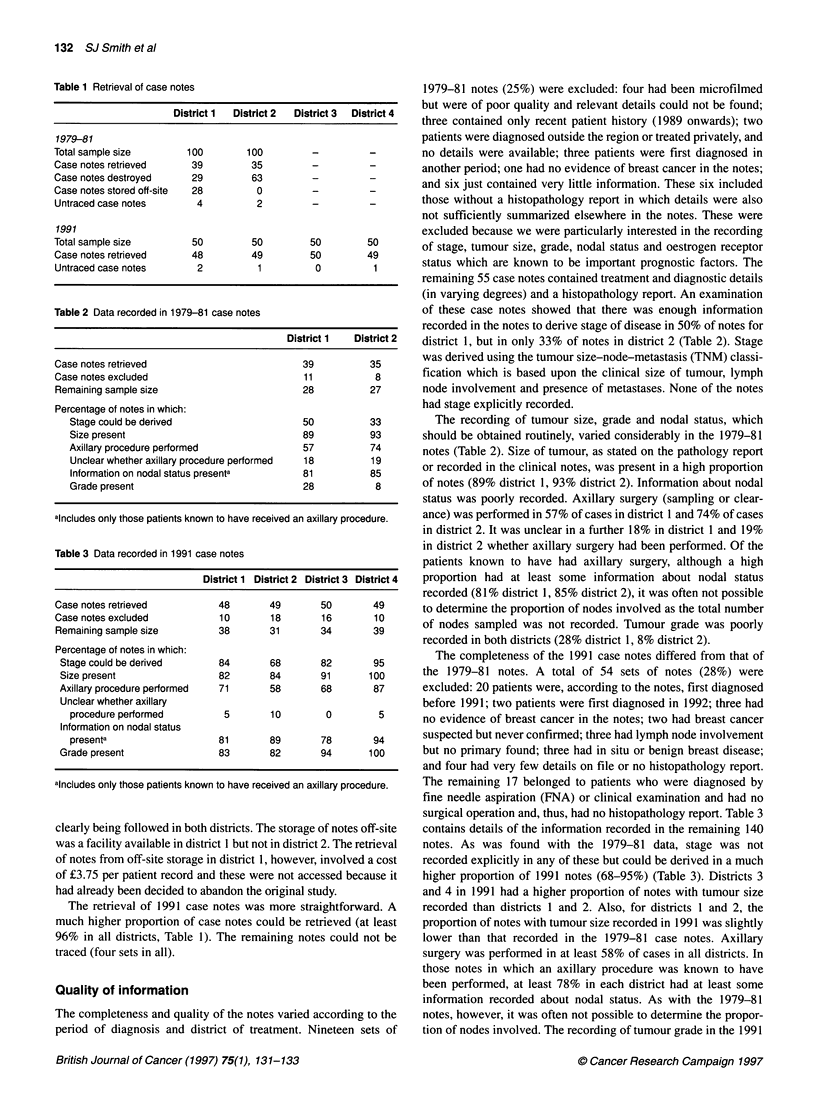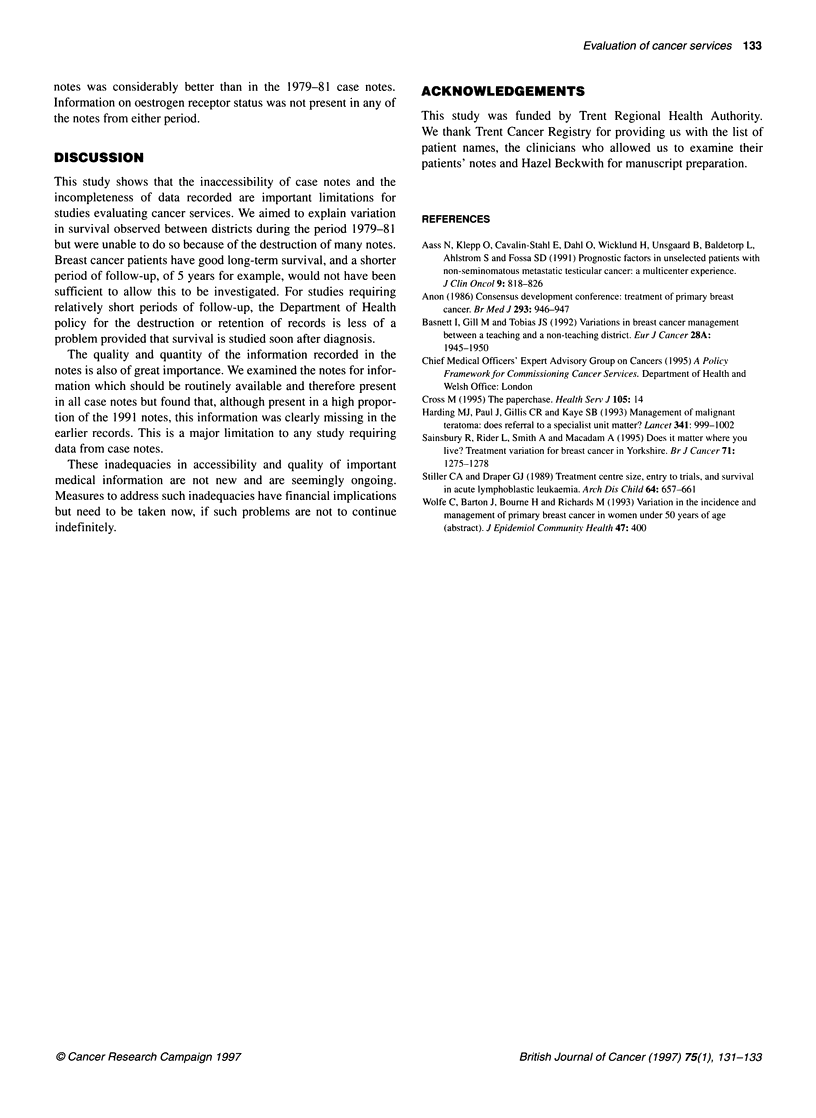# Continued inadequacies in data sources for the evaluation of cancer services.

**DOI:** 10.1038/bjc.1997.21

**Published:** 1997

**Authors:** S. J. Smith, K. R. Muir, J. L. Wolstenholme, K. G. Thornhill, A. Zamorski, K. Tolley, R. F. Logan, C. E. Chilvers

**Affiliations:** Trent Institute for Health Services Research, University of Nottingham Medical School, Queen's Medical Centre, UK.

## Abstract

There is a need to evaluate cancer services and provide a baseline on current treatment success and organization. This study shows that this process may be severely hindered by case note destruction or inaccessibility and incomplete information. This is an ongoing problem that needs to be addressed now.


British Journal of Cancer (1997) 75(1), 131–133
© 1997 Cancer Research Campaign

### Continued inadequacies in data sources for the evaluation of cancer services

**SJ Smith[1], KR Muir[2], JL Wolstenholme[1], KG Thornhill[1], A Zamorski[1], K Tolley[2], RFA Logan[2] and CED Chilvers[1,2,3]**

[1]Trent Institute for Health Services Research and [2]Department of Public Health Medicine and Epidemiology, University of Nottingham Medical School, Queen's Medical Centre, Nottingham NG7 2UH, UK; [3]Trent Cancer Registry, Weston Park Hospital NHS Trust, Whitham Road, Sheffield S10 2SJ, UK

**Summary** There is a need to evaluate cancer services and provide a baseline on current treatment success and organization. This study shows that this process may be severely hindered by case note destruction or inaccessibility and incomplete information. This is an ongoing problem that needs to be addressed now.

**Keywords:** cancer services; case note quality; case note accessibility

Attention has recently been directed towards the organization of cancer services in this country, following the suggestion that for several cancer sites there is regional and subregional variation in the quality of care provided (Basnett et al, 1992; Wolfe et al, 1993; Sainsbury et al, 1995). Both the media and, to a lesser extent, scientific groups have highlighted significant variations in survival according to where and how cases are diagnosed, and it has been suggested that patients) treated at specialist centres will have a better prognosis than those treated at smaller or district hospitals (Stiller and Draper, 1989; Aass et al, 1991; Harding et al, 1993).

The Calman report, *A Policy Framework for Commissioning Cancer Services*, recommended the establishment of a hierarchy of cancer centres and units in each National Health Service (NHS) region. The effectiveness of the proposed changes in the structure of patient care will clearly need to be evaluated, and audits for cancer services will be set up to do this. Current practices will also need to be evaluated to provide a baseline with which future services can be compared. These studies require information on prognostic factors, such as stage at presentation, treatment details, follow-up and outcome, so that meaningful comparisons can be made. The main source of information for studies of this kind is patient case notes, as these are currently the only source from which all the necessary data can be retrieved. Case note abstraction, however, can be a time-consuming process, and recent reports have suggested that hospital records are often inaccessible and fail to meet standards of legibility, accuracy, timeliness and completeness (Cross, 1995). The successful completion of such studies, therefore, may be considerably hindered by these problems. In this paper, we report our experience in retrieving case notes and abstracting data in a study set up to evaluate changes in the management of breast cancer in Trent Region. This study was abandoned before completion because of the low proportion and poor quality of case notes available.

*Received 4 March 1996*
*Revised 22 July 1996*
*Accepted 23 July 1996*

*Correspondence to:* SJ Smith

### PATIENTS AND METHODS

We examined the case notes of women with breast cancer from two time periods: 1979–81 and 1991. The former time period, 1979–81, was chosen as a result of earlier work using Trent Cancer Registry data that had shown marginally significant differences ($P$=0.08) in crude survival between districts for women diagnosed in this period. We had decided to investigate these differences further and to seek explanations for them using case note data. The two districts with the poorest survival and the two with the best survival were therefore identified, and a random sample of 100 breast cancer patients diagnosed in each of these four districts during the period 1979–81 was obtained from Trent Cancer Registry records. However, as the poor availability of case notes became apparent the number of districts at this time period was restricted to two, and the study evolved into an investigation of the quality of the available records. A random sample of 50 breast cancer patients diagnosed in 1991 in each of the four districts originally selected was taken for comparison. It had been hoped to see whether practices in the care of patients had changed since 1979–81 and also to determine the extent to which the King's Fund consensus guidelines (Anon, 1986) had been implemented. The notes that were obtained were examined for the details that had been required for the original study, i.e. diagnostic procedures, treatment, stage of disease and other prognostic factors, follow-up and outcome. Ethics committee approval was obtained in each of the four districts.

### RESULTS

### Retrieval of case records

In the two districts for which 1979–81 data were extracted, 35% and 39% of case notes were obtained, the remainder having been destroyed, stored off-site or become untraceable (Table 1). The large proportion of case notes that had been destroyed (46% overall) appeared to be a direct result of the policy issued by the Department of Health, stating that case notes can be destroyed 8 years after the last point of contact with a patient [circular HC (89)20]. This was issued as a strategy to alleviate case note storage problems within hospitals and, although not yet mandatory, was

**Table 1** Retrieval of case notes

| | District 1 | District 2 | District 3 | District 4 |
|---|---|---|---|---|
| *1979–81* | | | | |
| Total sample size | 100 | 100 | – | – |
| Case notes retrieved | 39 | 35 | – | – |
| Case notes destroyed | 29 | 63 | – | – |
| Case notes stored off-site | 28 | 0 | – | – |
| Untraced case notes | 4 | 2 | – | – |
| *1991* | | | | |
| Total sample size | 50 | 50 | 50 | 50 |
| Case notes retrieved | 48 | 49 | 50 | 49 |
| Untraced case notes | 2 | 1 | 0 | 1 |

**Table 2** Data recorded in 1979–81 case notes

| | District 1 | District 2 |
|---|---|---|
| Case notes retrieved | 39 | 35 |
| Case notes excluded | 11 | 8 |
| Remaining sample size | 28 | 27 |
| Percentage of notes in which: | | |
| Stage could be derived | 50 | 33 |
| Size present | 89 | 93 |
| Axillary procedure performed | 57 | 74 |
| Unclear whether axillary procedure performed | 18 | 19 |
| Information on nodal status present[a] | 81 | 85 |
| Grade present | 28 | 8 |

[a]Includes only those patients known to have received an axillary procedure.

**Table 3** Data recorded in 1991 case notes

| | District 1 | District 2 | District 3 | District 4 |
|---|---|---|---|---|
| Case notes retrieved | 48 | 49 | 50 | 49 |
| Case notes excluded | 10 | 18 | 16 | 10 |
| Remaining sample size | 38 | 31 | 34 | 39 |
| Percentage of notes in which: | | | | |
| Stage could be derived | 84 | 68 | 82 | 95 |
| Size present | 82 | 84 | 91 | 100 |
| Axillary procedure performed | 71 | 58 | 68 | 87 |
| Unclear whether axillary procedure performed | 5 | 10 | 0 | 5 |
| Information on nodal status present[a] | 81 | 89 | 78 | 94 |
| Grade present | 83 | 82 | 94 | 100 |

[a]Includes only those patients known to have received an axillary procedure.

clearly being followed in both districts. The storage of notes off-site was a facility available in district 1 but not in district 2. The retrieval of notes from off-site storage in district 1, however, involved a cost of £3.75 per patient record and these were not accessed because it had already been decided to abandon the original study.

The retrieval of 1991 case notes was more straightforward. A much higher proportion of case notes could be retrieved (at least 96% in all districts, Table 1). The remaining notes could not be traced (four sets in all).

### Quality of information

The completeness and quality of the notes varied according to the period of diagnosis and district of treatment. Nineteen sets of 1979–81 notes (25%) were excluded: four had been microfilmed but were of poor quality and relevant details could not be found; three contained only recent patient history (1989 onwards); two patients were diagnosed outside the region or treated privately, and no details were available; three patients were first diagnosed in another period; one had no evidence of breast cancer in the notes; and six just contained very little information. These six included those without a histopathology report in which details were also not sufficiently summarized elsewhere in the notes. These were excluded because we were particularly interested in the recording of stage, tumour size, grade, nodal status and oestrogen receptor status which are known to be important prognostic factors. The remaining 55 case notes contained treatment and diagnostic details (in varying degrees) and a histopathology report. An examination of these case notes showed that there was enough information recorded in the notes to derive stage of disease in 50% of notes for district 1, but in only 33% of notes in district 2 (Table 2). Stage was derived using the tumour size–node–metastasis (TNM) classification which is based upon the clinical size of tumour, lymph node involvement and presence of metastases. None of the notes had stage explicitly recorded.

The recording of tumour size, grade and nodal status, which should be obtained routinely, varied considerably in the 1979–81 notes (Table 2). Size of tumour, as stated on the pathology report or recorded in the clinical notes, was present in a high proportion of notes (89% district 1, 93% district 2). Information about nodal status was poorly recorded. Axillary surgery (sampling or clearance) was performed in 57% of cases in district 1 and 74% of cases in district 2. It was unclear in a further 18% in district 1 and 19% in district 2 whether axillary surgery had been performed. Of the patients known to have had axillary surgery, although a high proportion had at least some information about nodal status recorded (81% district 1, 85% district 2), it was often not possible to determine the proportion of nodes involved as the total number of nodes sampled was not recorded. Tumour grade was poorly recorded in both districts (28% district 1, 8% district 2).

The completeness of the 1991 case notes differed from that of the 1979–81 notes. A total of 54 sets of notes (28%) were excluded: 20 patients were, according to the notes, first diagnosed before 1991; two patients were first diagnosed in 1992; three had no evidence of breast cancer in the notes; two had breast cancer suspected but never confirmed; three had lymph node involvement but no primary found; three had in situ or benign breast disease; and four had very few details on file or no histopathology report. The remaining 17 belonged to patients who were diagnosed by fine needle aspiration (FNA) or clinical examination and had no surgical operation and, thus, had no histopathology report. Table 3 contains details of the information recorded in the remaining 140 notes. As was found with the 1979–81 data, stage was not recorded explicitly in any of these but could be derived in a much higher proportion of 1991 notes (68–95%) (Table 3). Districts 3 and 4 in 1991 had a higher proportion of notes with tumour size recorded than districts 1 and 2. Also, for districts 1 and 2, the proportion of notes with tumour size recorded in 1991 was slightly lower than that recorded in the 1979–81 case notes. Axillary surgery was performed in at least 58% of cases in all districts. In those notes in which an axillary procedure was known to have been performed, at least 78% in each district had at least some information recorded about nodal status. As with the 1979–81 notes, however, it was often not possible to determine the proportion of nodes involved. The recording of tumour grade in the 1991

© Cancer Research Campaign 1997

notes was considerably better than in the 1979–81 case notes. Information on oestrogen receptor status was not present in any of the notes from either period.

### DISCUSSION

This study shows that the inaccessibility of case notes and the incompleteness of data recorded are important limitations for studies evaluating cancer services. We aimed to explain variation in survival observed between districts during the period 1979–81 but were unable to do so because of the destruction of many notes. Breast cancer patients have good long-term survival, and a shorter period of follow-up, of 5 years for example, would not have been sufficient to allow this to be investigated. For studies requiring relatively short periods of follow-up, the Department of Health policy for the destruction or retention of records is less of a problem provided that survival is studied soon after diagnosis.

The quality and quantity of the information recorded in the notes is also of great importance. We examined the notes for information which should be routinely available and therefore present in all case notes but found that, although present in a high proportion of the 1991 notes, this information was clearly missing in the earlier records. This is a major limitation to any study requiring data from case notes.

These inadequacies in accessibility and quality of important medical information are not new and are seemingly ongoing. Measures to address such inadequacies have financial implications but need to be taken now, if such problems are not to continue indefinitely.

### ACKNOWLEDGEMENTS

This study was funded by Trent Regional Health Authority. We thank Trent Cancer Registry for providing us with the list of patient names, the clinicians who allowed us to examine their patients' notes and Hazel Beckwith for manuscript preparation.

### REFERENCES

Aass N, Klepp O, Cavalin-Stahl E, Dahl O, Wicklund H, Unsgaard B, Baldetorp L, Ahlstrom S and Fossa SD (1991) Prognostic factors in unselected patients with non-seminomatous metastatic testicular cancer: a multicenter experience. *J Clin Oncol* **9**: 818–826

Anon (1986) Consensus development conference: treatment of primary breast cancer. *Br Med J* **293**: 946–947

Basnett I, Gill M and Tobias JS (1992) Variations in breast cancer management between a teaching and a non-teaching district. *Eur J Cancer* **28A**: 1945–1950

Chief Medical Officers' Expert Advisory Group on Cancers (1995) *A Policy Framework for Commissioning Cancer Services*. Department of Health and Welsh Office: London

Cross M (1995) The paperchase. *Health Serv J* **105**: 14

Harding MJ, Paul J, Gillis CR and Kaye SB (1993) Management of malignant teratoma: does referral to a specialist unit matter? *Lancet* **341**: 999–1002

Sainsbury R, Rider L, Smith A and Macadam A (1995) Does it matter where you live? Treatment variation for breast cancer in Yorkshire. *Br J Cancer* **71**: 1275–1278

Stiller CA and Draper GJ (1989) Treatment centre size, entry to trials, and survival in acute lymphoblastic leukaemia. *Arch Dis Child* **64**: 657–661

Wolfe C, Barton J, Bourne H and Richards M (1993) Variation in the incidence and management of primary breast cancer in women under 50 years of age (abstract). *J Epidemiol Community Health* **47**: 400

© Cancer Research Campaign 1997